# Supplementary material for: Monolithically integrated, broadband, high-efficiency silicon nitride-on-silicon waveguide photodetectors in a visible-light integrated photonics platform
Source: Nat Commun. 2022 Oct 26;13:6362. doi: 10.1038/s41467-022-34100-3 (PMC9606291; doi:10.1038/s41467-022-34100-3)
Supplement: Supplementary file 1 — Supplementary information [file 41467_2022_34100_MOESM1_ESM.pdf]

Monolithically integrated, broadband, high-efficiency silicon nitride-on-silicon waveguide photodetectors in a visible-light integrated photonics platform: Supplementary information

Yiding Lin[1], Zheng Yong[2], Xianshu Luo[3], Saeed Sharif Azadeh[1],
Jared Mikkelsen[1], Ankita Sharma[1,2], Hong Chen[1], Jason C. C. Mak[2],
Patrick Guo-Qiang Lo[3], Wesley D. Sacher[1], and Joyce K. S. Poon[1,2]

[1]*Max Planck Institute of Microstructure Physics, Weinberg 2, 06120 Halle, Germany*

[2]*Department of Electrical and Computer Engineering,*
University of Toronto, 10 King's College Road,
Toronto, Ontario M5S 3G4, Canada and

[3]*Advanced Micro Foundry Pte Ltd, 11 Science Park Road,*
Singapore Science Park II, 117685, Singapore

S1. VIA SEPARATION AND SI MESA HEIGHT

We computed the SiN waveguide propagation loss using a finite difference eigenmode (FDE) solver (Lumerical MODE Solutions) to set the Al via separation and the Si mesa height in the PD design. Figure S1(a) shows the waveguide loss for the TM_0 and TE_0 modes at λ = 405, 488, 532 and 640 nm as a function of SiN-Via1 gap. The inset shows the schematic for the SiN waveguide and Via1 parameters used in the calculation. The Si substrate is not included to isolate the effect of via. The loss due to the Al via is < 0.02 dB/cm for all the wavelengths at a gap of 6 µm, which is the minimum separation in the PDs presented.

Figure S1(b) shows the waveguide loss as a function of Si mesa height. Here, the waveguide widths (W_{gw}) are 380 nm at λ = 405, 488 and 532 nm, and 500 nm at λ = 640 nm. For W_{gw} = 380 nm, the loss is $< 10^{-6}$ dB/cm at the mesa height of 2.85 μm for wavelengths up to 532 nm. For longer wavelengths, the loss can be less than 4×10^{-4} dB/cm for W_{gw} = 500 nm up to λ = 640 nm. Therefore, the Si mesa height was set to 2.85 μm with W_{gw} = 500 nm for a low propagation loss up to λ = 640 nm, and we used λ = 514 nm for the tunable microring design and measurement with W_{gw} = 380 nm.

These calculations used a background index of 1.46 and metal boundary conditions. At the boundary, at $\lambda = 640$ nm, the electric field magnitude was 10^{-7} of the maximum.

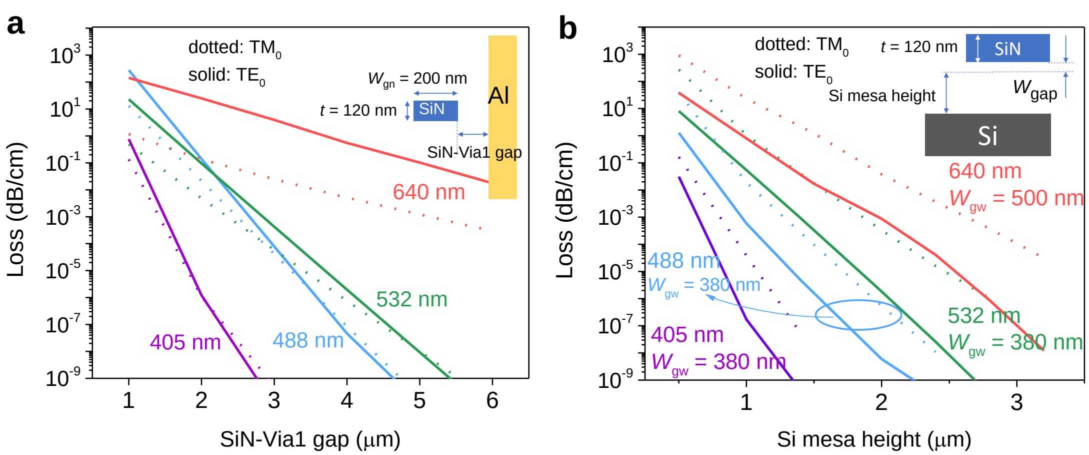

FIG. S1. Calculated SiN waveguide loss as a function of (a) SiN-Via1 gap and (b) Si mesa height at wavelengths of 405, 488, 532 and 640 nm

S2. EDGE COUPLING AND WAVEGUIDE PROPAGATION LOSS

Figure S2 show the measured edge coupler and waveguide propagation loss. Cleaved single-mode fibers coupled light into and out of the respective test structures on chip [1, 2]. The higher coupling loss at short wavelengths is due to the lower mode overlap between the fiber and on-chip edge coupler [2]. The lower coupling loss for TM relative to TE is due to the reduced optical confinement of the fundamental TM mode in the edge coupler and correspondingly a better mode overlap with the fiber [1]. The measurement error is due to the variability in the input/output coupling and waveguide loss. We have also observed similar loss variability on the wafers in [2].

S3. PD LINEARITY AND PHOTOCURRENTS IN PN VS. PIN JUNCTIONS

Fig S3 shows the effective photocurrent is a linear function of the input power of the 50-µm long PN PD. Figure S4 shows that the observed photocurrents of the PN and PIN PDs are nearly identical.

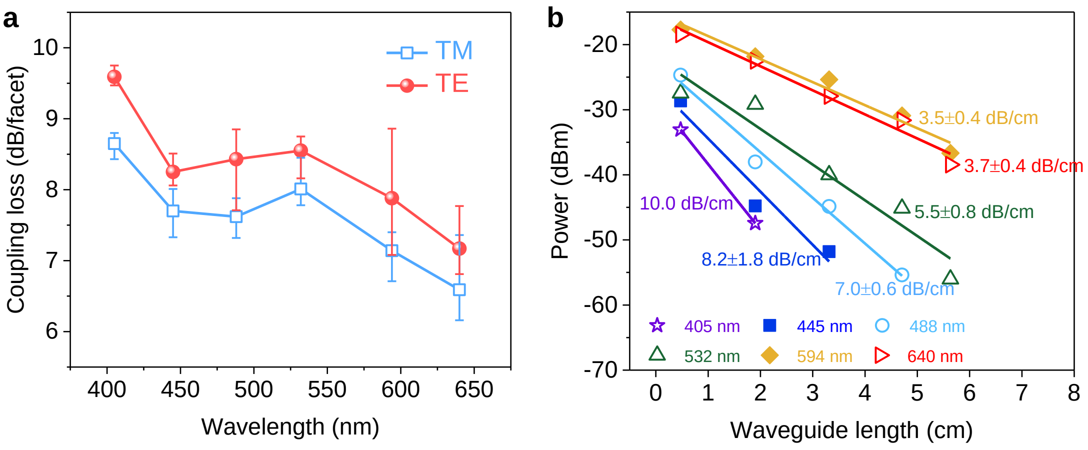

FIG. S2. (a) Measured coupling loss of SiN tapered edge couplers (from 3 chips far apart on wafer). The error bars show the maximum and minimum quantities measured for the 3 devices, and the data point shows the average. (b) Measured propagation loss of routing SiN waveguides (width = 520 nm) at TE polarized mode.

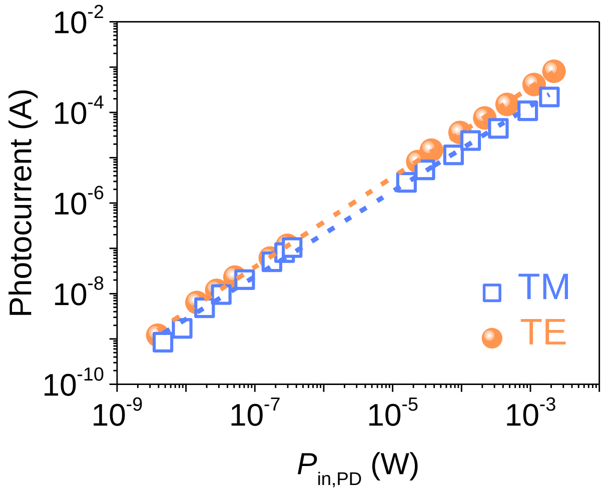

FIG. S3. I_{eph} for a 50-µm long PN device as a function of $P_{in,PD}$ from $\sim 10^{-9}$ to 10^{-3} W at $\lambda = 488$ nm. A good linearity for both TE and TM polarizations was observed within this range of power. Responsivities: TE: 0.33 ± 0.05 A/W, TM: 0.30 ± 0.05 A/W.

S4. CALCULATION OF η_{mode}

Figures S5(a) and (b) show, respectively, the TM_0 mode profiles at $\lambda = 488$ nm in the narrowed part of the SiN waveguide (W_{gn}) and in the SiN-on-Si mesa region. The waveguide

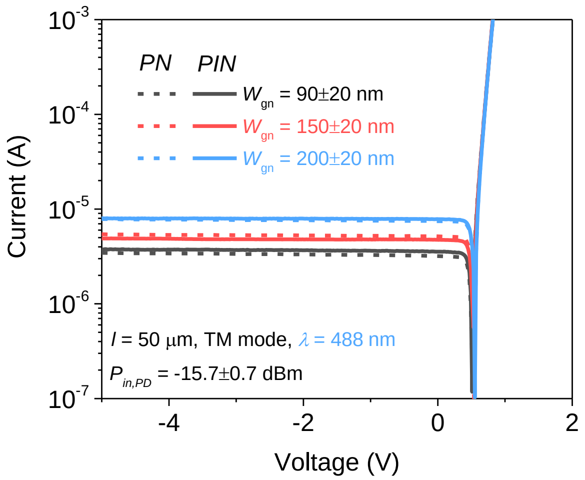

FIG. S4. Measured photocurrents of PN and PIN PDs at different W_{gn} (measured from TEM images) as a function of applied voltage at $\lambda = 488$ nm.

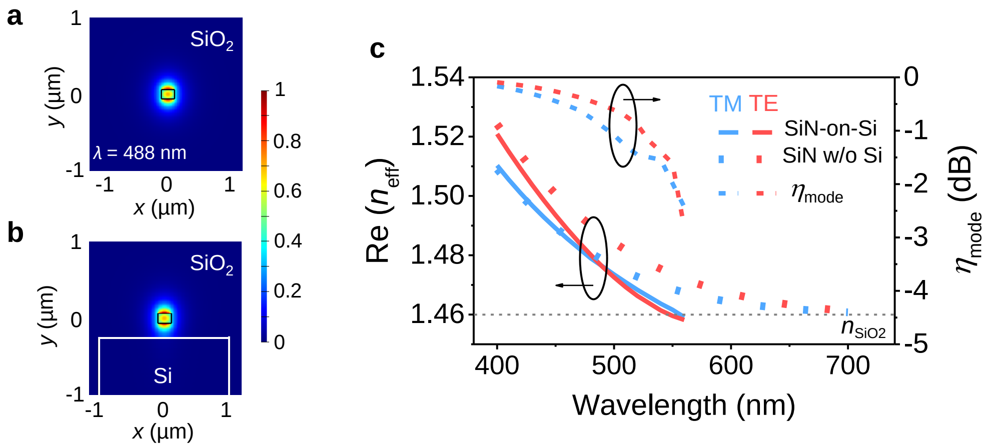

FIG. S5. TM_0 mode profiles at $\lambda = 488$ nm in the SiN waveguide ($W_{gn} = 200$ nm) (a) without and (b) with the Si mesa. (c) Simulated Re(n_{eff}) of SiN without (as in (a)) and with (as in (b)) the Si underneath, as well as their corresponding mode mismatch loss (η_{mode}, in dB), as a function of wavelength.

dimensions used in the calculation are the average values from the TEM images $W_{gn} = 200$ nm, $t = 120$ nm, and $W_{gap} = 190$ nm). Figure S5(c) shows the computed mode mismatch loss (η_{mode}) determined from the corresponding mode overlap using a finite difference eigenmode (FDE) solver (Lumerical MODE Solutions). The refractive indices used for SiN and SiO_2 cladding were 1.82 and 1.46, respectively, for all wavelengths in the calculation (400-700

nm). The Si material properties were imported from the default database "Si (Silicon) - Palik". The width of Si was 2 µm in the simulation and the structure was extended down beyond the simulation region to mimic bulk Si. Extending the Si width leads to a negligible change on η_{mode}. The 2-D simulation region on the cross-sectional plane of the waveguide spans 5 µm in width and 4 µm in height, with the SiN waveguide sharing the same center point. Metal boundaries were used at the boundaries of the simulation region. Mode overlap calculation was performed between the eigenmode profiles with only the SiN and additionally with the Si underneath for η_{mode}. Due to the similarity of the mode profiles (Fig. S5(a) and (b)), the η_{mode} is < -2 dB for $\lambda \in [400, 500]$ nm for both TE and TM polarized light (Fig. S5(c)). The computation was not extended to longer wavelengths since the real part of SiN-on-Si effective indices ($\mathrm{Re}(n_{eff})$) dropped below the cladding index (1.46) at $\lambda > 550$ nm (Fig. S5(c)). The mode mismatch loss increases at longer wavelengths due to the reduced waveguide confinement factor [3].

S5. SIMULATION OF EQE

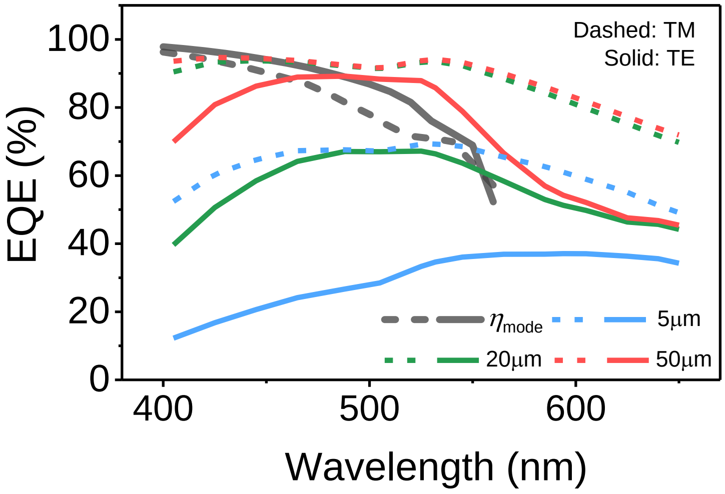

FIG. S6. Simulated EQE vs. device length (l , in Fig. 1(d), $l = 5, 20, 50$ µm). η_{mode} (from Fig. S5(c)) is included for comparison.

The simulation of the PD EQE was performed using the 3D finite-difference time-domain method (Lumerical FDTD). Material property settings were identical to that in Section S4. The Si mesa had a width of 2 µm, and the Si thickness was 5 µm for $\lambda \leq 532$ nm and 9 µm

for $\lambda > 532$ nm. The Si thickness leads to an error of < 1% in the total absorbed optical power for a 50 µm long device. Average waveguide dimensions $W_{gn} = 200$ nm, $t = 120$ nm, and $W_{gap} = 190$ nm) from the TEM images were used. Perfectly matched layers (PMLs) were used at the simulation region boundaries. To calculate the EQE, the total absorbed power in Si, given by the absorbed power density, $-\frac{1}{2}\omega \text{Im}(\epsilon)|E|^2$, was integrated over the Si region and normalized to the input optical power. This calculation assumes a perfect internal quantum efficiency, i.e., all absorbed photons are converted to carriers collected by the electrodes. The EQE at device lengths (l, Fig. 1(b)) of 5, 10, 20, 30, and 50 µm, from operating wavelengths of 400 to 700 nm were calculated. The cross-sectional area of the injected mode source was kept constant at a width of 4 µm and height of 3.8 µm. For device lengths < 50 µm, the simulation contained the 50 µm-long device but the power density was integrated to the device length. These simulation settings were applied to all subsequent EQE calculations here. Fig. S6 shows the calculated EQE at $l = 5$, 20 and 50 µm, where η_{mode} (Fig. S5(c)) was also included for comparison. A device with l= 50 µm approached the EQE limited by η_{mode} between the input SiN waveguide and the SiN-on-Si region. The simulation results are generally higher than the measurements (Fig. 2(d)), since the simulations assume a perfect internal quantum efficiency and neglect losses. We observed negligible EQE difference between PN and PIN devices (Fig. S4).

S6. ABSORPTION MECHANISM ANALYSIS

TABLE S1. Fitted parameters for EQE vs. l (Eq. S1) at different visible wavelengths.

Wavelength (nm)	A		C		$A+C$		$\alpha_{coupling}$ (µm^{-1})	
	TM	TE	TM	TE	TM	TE	TM	TE
405	-0.97	-0.94	0.94	0.94	-0.03	-0.00	0.17	0.03
445	-1.04	-0.94	0.94	0.95	-0.10	0.01	0.25	0.05
488	-1.13	-0.91	0.92	0.93	-0.22	-0.02	0.30	0.06
532	-0.88	-0.73	0.94	0.90	0.06	0.17	0.25	0.06
594	-0.60	-0.31	0.83	0.54	0.23	0.23	0.19	0.12
640	-0.56	-0.28	0.73	0.47	0.17	0.18	0.19	0.19

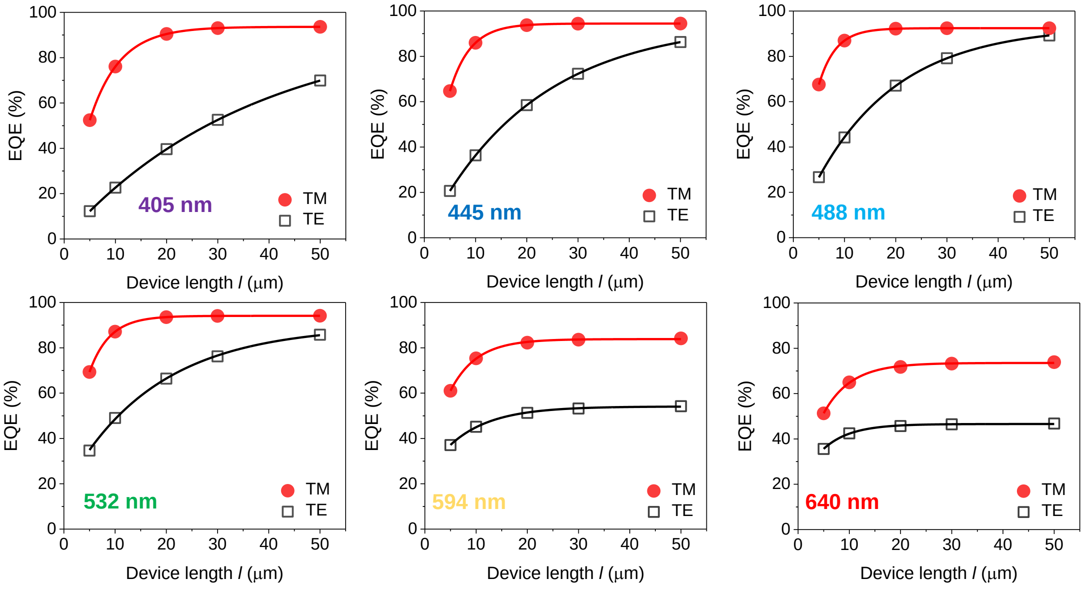

FIG. S7. Simulated EQE as a function of device length l at different wavelengths. The data points are fitted with an exponential and the extracted parameters are tabulated in Table S1.

We choose the 6 wavelengths (405, 445, 488, 532, 594 and 640 nm) used in the measurements to study the light absorption mechanisms in Si. Figure S7 shows the simulated EQE as a function of device length l at these wavelengths. First, we fitted the EQE to [4]

$$\text{EQE} = A\text{e}^{-\alpha_{coupling} l} + C, \tag{S1}$$

where A and C are constants and $\alpha_{coupling}$ is the coupling efficiency into Si. $A + C$ is the theoretical EQE as $l \to 0$, and C is the maximum achievable EQE as $l \to \infty$. The solid lines in Fig. S7 are the fits and the fitting parameters are tabulated in Table S1. Generally, for $\lambda \geq 532$ nm where $(A + C) > 0$ and $A + C$ increases with wavelength, a fraction of the launched light in the SiN waveguide is absorbed in the Si at the front facet of the mesa. The trend agrees with the cross-sectional $|E|$ profiles in Fig. S8, where $|E|$ at the input Si mesa facet increases in amplitude with increasing wavelength (indicated by arrows). The TM polarized mode at longer wavelengths is more strongly scattered into Si since the input Si facet represents a greater perturbation to the mode. The EQE for $\lambda \geq 532$ nm is due to a combined effect of the absorption of scattered light and the evanescent coupling between SiN and Si, while the EQE at the shorter wavelengths is mainly achieved by evanescent coupling. $\alpha_{coupling}$ monotonically increases with wavelength for the TE polarization, but shows a peak at $\lambda = 488$ nm for the TM polarization. This is a result of the increasing mode overlap

with the Si in conjunction with the lower Si absorption as the wavelength increases. As the wavelength increases to $\sim$ 532 nm, the TM mode is cut off first, leading to a reduction of $\alpha_{coupling}$.

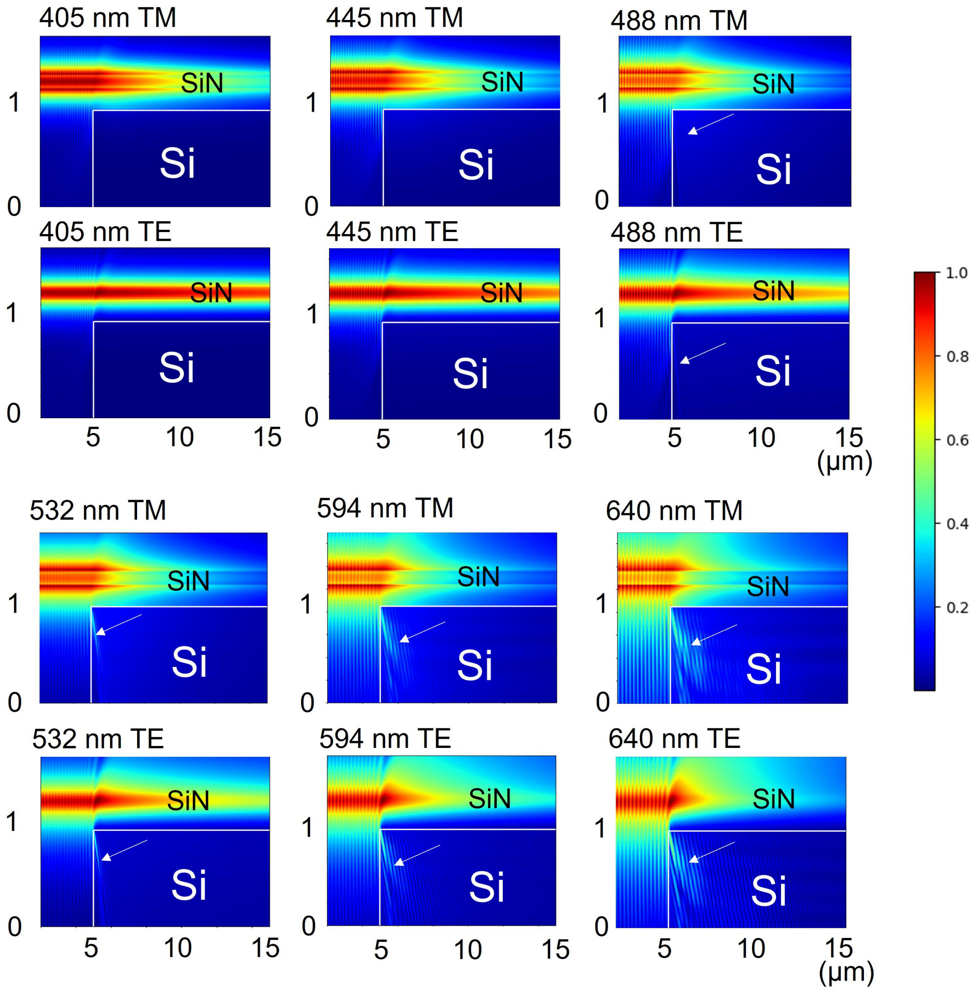

FIG. S8. Cross-sectional $|E|$ profiles at different visible wavelengths. The cladding is SiO_2. Light scattering into Si is more significant at longer wavelengths since the input mode is less confined in the SiN waveguide. The arrows are not included in the figures at $\lambda = 405$ and 445 nm due to weak scattering.

S7. RESPONSIVITY VS. DEVICE LENGTH

Figure S9 shows the measured responsivity of PIN PDs vs. l at several wavelengths. The error bars show the maximum and minimum quantities observed across 3 chips, and the

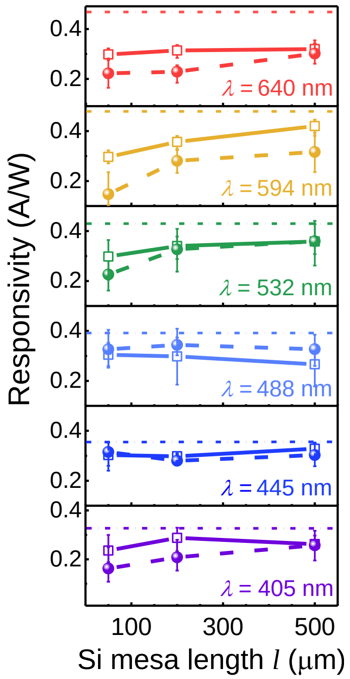

FIG. S9. Measured responsivity of PIN devices as a function of device length (l) at several wavelengths (TM: solid curves; TE: dashed curves). Each data point was averaged from the measurements of 3 chips far apart on the wafer, and the error bars show the maximum and minimum quantities. The dotted lines indicate the theoretical maximum responsivities. The responsivity increased by $< 30\%$ for devices with $l > 50$ µm.

data point shows the average.

S8. EQE ENHANCEMENT FOR TE AT $\lambda = 405$ NM BY NARROWING SIN WIDTH (W_{gn})

As seen in Fig. 2(d), the EQE decreased at $\lambda \sim 450$ nm for TE due to the reduced coupling coefficient ($\alpha_{coupling}$) between the SiN waveguide and Si (see Section S6). To enhance $\alpha_{coupling}$, we can narrow W_{gn} to increase the rate of power transfer from SiN into Si. Figure S10 shows this effect of W_{gn} at $\lambda = 405$ nm for the TE polarized mode as an example. As expected, both the measured and simulated EQE exhibited a consistent increase with W_{gn} decreasing down to ∼150 nm. A further decrease of W_{gn} resulted in a drastic drop on EQE, likely due to the low mode confinement in SiN causing power scattering into ambient. The

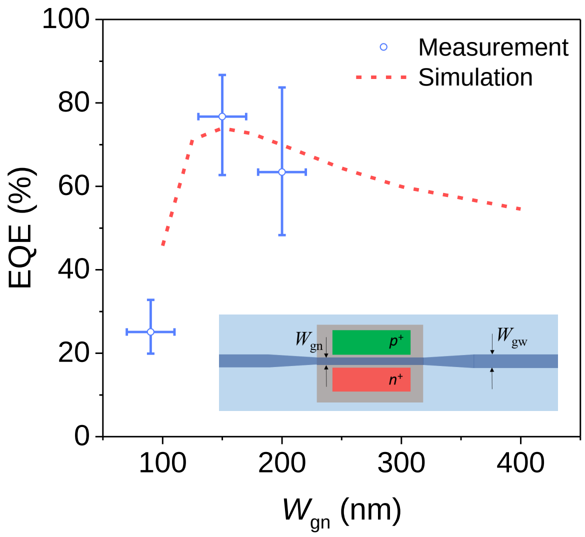

FIG. S10. Measured and simulated EQE for the SiN-on-Si photodiodes as a function of W_{gn} at $\lambda = 405$ nm for the TE polarized mode. The vertical error bars show the maximum and minimum quantities measured for the 3 devices; the horizontal error bars indicate the possible width variation; and the data point shows the average. Narrowing the W_{gn} up to 150 nm enhances the evanescent light penetration into Si and consequently the EQE.

simulation indicates a total of ∼19% EQE enhancement by narrowing W_{gn} from 400 to 150 nm.

S9. EFFECT OF DEVICE DESIGN DIMENSIONS: SIMULATION STUDY

The EQE was also simulated (see procedures in Section S5) as a function of some key device dimensions. Figure S11(a) shows a schematic of the device with these parameters. Figure S11(b) shows the effect of Si thickness t_{Si} on EQE. In the simulations, a buried oxide (SiO_2) layer of thickness t_{BOX} was added under the Si such that the $t_{Si} + t_{BOX}$ was 5 µm for $\lambda \leq 532$ nm and 9 µm for $\lambda > 532$ nm. At $\lambda = 640$ nm, the EQE fluctuates as a function of t_{Si} and flattened as t_{Si} increases. This may be explained by the dependence of the absorption on the phase-matching between the SiN waveguide mode and the slab modes in the thin Si layer. As t_{Si} increases and for shorter wavelengths, more modes within Si are supported, which leads to an EQE that is less sensitive to t_{Si}. At longer wavelengths, Si also has a lower absorption coefficient, which results in a larger penetration depth into Si of the leaky SiN mode. The Si mesa in our PD design (with a height of 2.85 µm) results in an

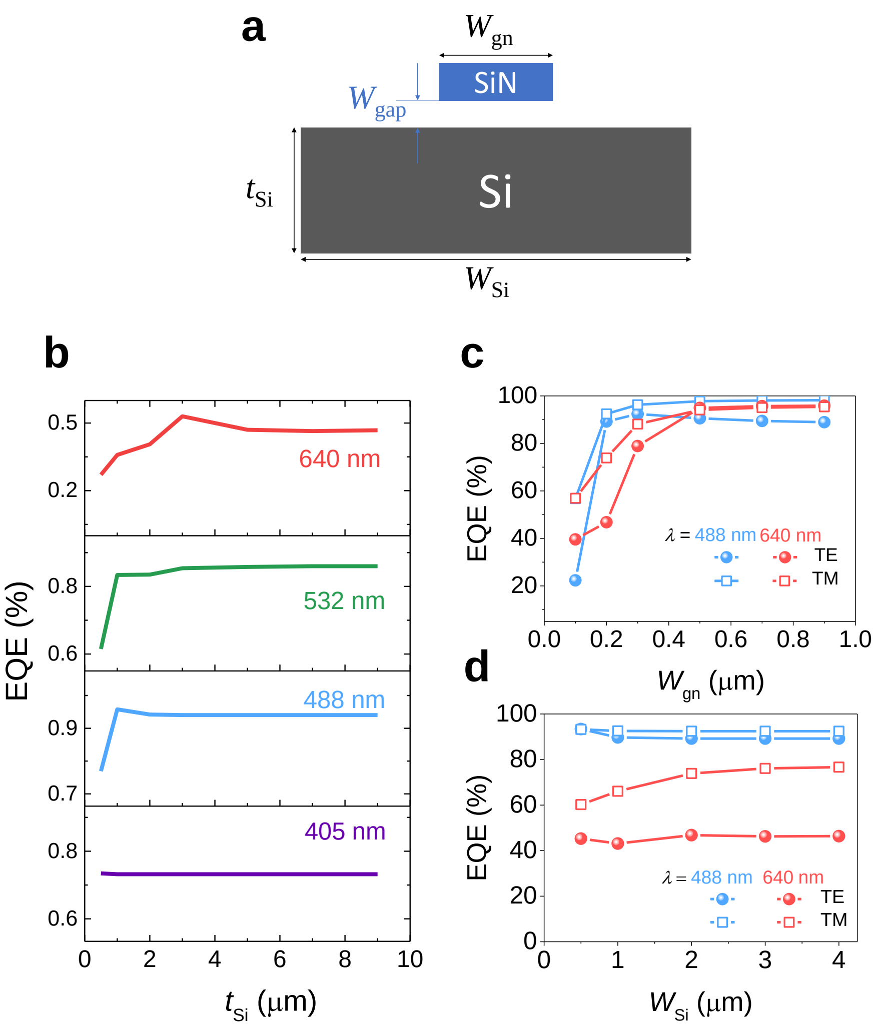

FIG. S11. EQE vs. Si thickness, SiN and Si widths. (a) Schematic of SiN-on-Si PD showing the parameters being studied. (b-d) Simulated EQE as a function of (b) Si thickness (t_{Si}) at different visible wavelengths, (c) SiN width (W_{gn}) and (d) Si mesa width (W_{Si}) at λ = 488 and 640 nm. The device length (l) used in the simulations is 50 µm. When held constant, the other geometrical parameters are: W_{Si} = 2 µm; W_{gn} = 200 nm; t = 120 nm; W_{gap} = 190 nm; and t_{Si} = 5 µm for $\lambda \leq 532$ nm and 9 µm for $\lambda > 532$ nm.

EQE that is fairly insensitive to Si thickness.

Figure S11(c)-(d) show the sensitivity of the EQE to the SiN width (W_{gn}) and Si mesa width (W_{Si}). The EQE is sensitive to W_{gn} near 200 nm for the two illustrated wavelengths, because the waveguide modes are near or at cut-off (Fig. S5). Nonetheless, we chose W_{gn} = 200 nm to maintain a high EQE at the short wavelengths (> 60% for λ = 405 nm).

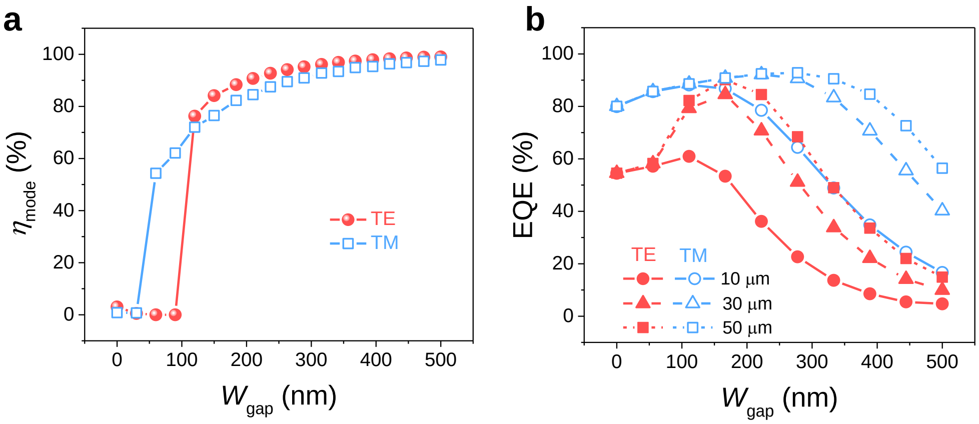

FIG. S12. The effect of W_{gap} on (a) η_{mode} and (b) EQE at $\lambda = 488$ nm. The device lengths in (b) are 10, 30 and 50 µm, and other design parameters are: $W_{Si} = 2$ µm; $W_{gn} = 200$ nm; $t = 120$ nm; and $t_{Si} = 10$ µm. A W_{gap} of ~110-220 nm optimizes the EQE.

The EQE is insensitive to $W_{Si} \gtrsim 500$ nm.

Figure S12 shows the effect of W_{gap}, the interlayer spacing, on the mode mismatch loss and EQE. Due to the loss of mode confinement in SiN in the presence of Si, $\eta_{mode} \rightarrow 0$ as $W_{gap} \rightarrow 0$. $\eta_{mode} \rightarrow 1$ as the Si is separated from the SiN. The EQE peaks at W_{gap} of $\sim 110 - 220$ nm for both TE and TM polarizations. The EQE decrease is due to the mode mismatch at small W_{gap} and lower evanescent coupling at large W_{gap}. To achieve both a high EQE and speed (i.e., at a short device length), a general rule can be to first determine the maximum length that can be used, and then choose a W_{gap} to maximize the EQE.

S10. JUNCTION CAPACITANCE AND CONTACT RESISTANCE EXTRACTION FOR 3-DB OE BANDWIDTH CALCULATION

The frequency response of PN junction-based photodetectors is determined by both the carrier transit time across the junction and the resistance-capacitance (RC) delay of the device [5]. The carrier transit involves both the drift and diffusion processes and is thus difficult to estimate, while the RC delay estimation is relatively easier, as the device contact resistance R and junction capacitance C_j can be directly extracted from measurements. Calculating the RC-limited frequency response helps to understand the dominant factors determining the device 3-dB OE bandwidth. A higher reverse bias ($|V_r|$) leads to both a

lower C_j and a shorter carrier transit time, which both contribute to a higher 3-dB OE bandwidth (i.e., shorter FWHM of impulse response seen in Fig. 2(e)). In this section, we show the extracted R and C_j values, estimates of RC-limited frequency response, and the comparison with our measurements.

A. Junction capacitance extraction

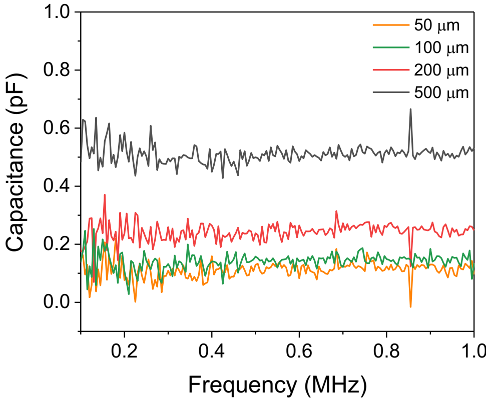

FIG. S13. An example plot of measured capacitance (C) for PN devices with different lengths at -1 V, as a function of sweeping frequency.

Fig. S13 shows that the capacitance measured by an impedance analyzer is independent of the sweeping frequency at all device lengths. The measurement results have been averaged to reduce noise, and the obtained averages can be linearly fit and extrapolated (see Eq. S2) at different reverse biases to extract C_j and parasitic capacitance C_p (Figs. S14(a), (b)). C_j scales linearly with device length l, while C_p can be treated as a constant as we applied identical metal wire and contact pad design for all devices. The measured capacitance, C is given by

$$C = C_{sj} \cdot l + C_p, \tag{S2}$$

where C_{sj} is the junction capacitance per unit length, and $C_j = C_{sj} \cdot l$. Hence, we can extract C_j and C_p from the slopes and y-intercepts of the linear fittings and extrapolations, respectively.

From the extrapolation, $C_p = 70 \pm 21$ and 55 ± 17 fF for PIN and PN devices, respectively, throughout the measured reverse biases. Figures S14(c) and (d) show the extracted C_{sj} as a

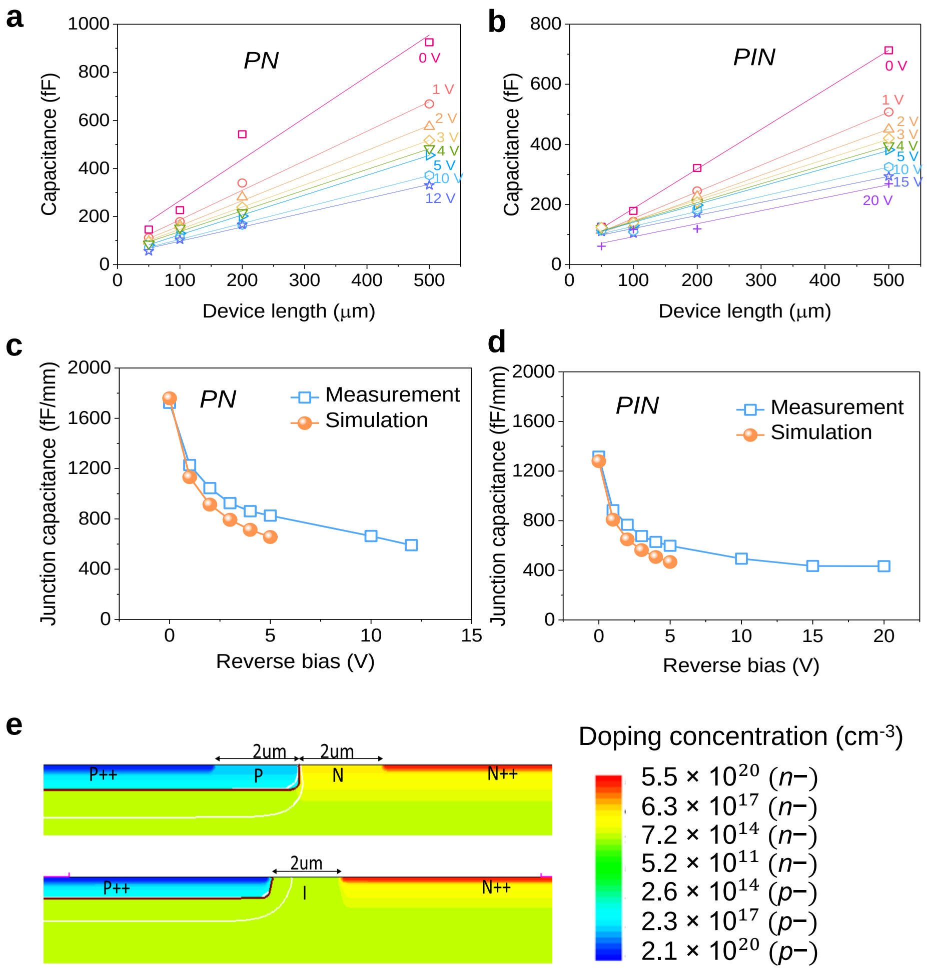

FIG. S14. Average measured capacitance (C) for (a) PN and (b) PIN devices as a function of device length at different reverse biases. The corresponding linear fittings resulted in device C_{sj} (slope) and C_p (y-intercept). The respective C_{sj} are shown in (c) and (d). (e) Simulated cross-sectional doping profiles for the PN and PIN junctions.

function of reverse bias for the PN and PIN device, respectively. The data agrees well with that calculated from the technology computer-aided design (Sentaurus TCAD) simulations of an applied bias of 0 to -5 V, using the implantation and rapid-thermal annealing (RTA) conditions in the fabrication. The Si thickness in the simulation was 4 μm to mimic bulk Si. The resulting doping profiles from the TCAD simulations are shown in Fig. S14(e).

B. Contact resistance extraction

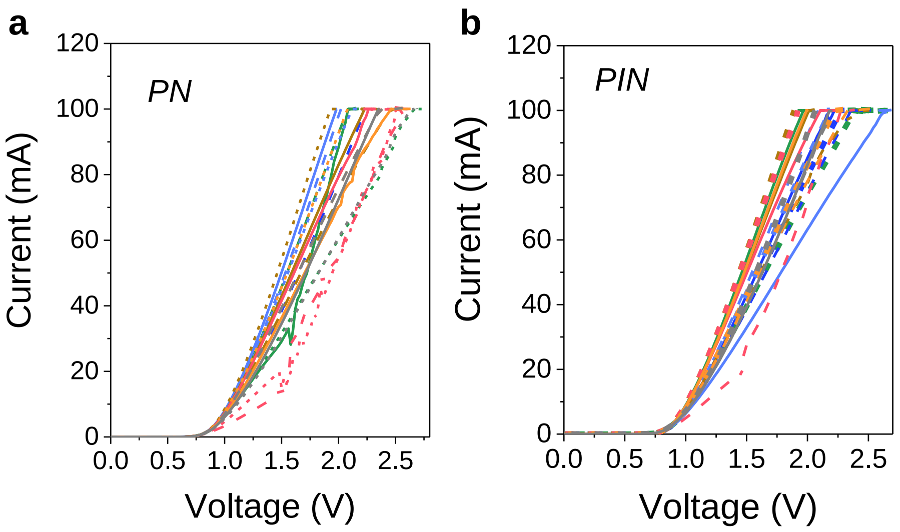

FIG. S15. Summary of 50-µm long (a) PN and (b) PIN device $I - V$ characteristics (linear scale) at forward bias for the extraction of contact resistance. In total, 21 devices from 7 chips were measured for each device type, which are located uniformly throughout the wafer.

Figure S15 summarizes the $I - V$ characteristics of the 50-µm long PN and PIN devices in forward bias. Beyond the voltage of 1.5 V, most of the curves exhibit a linear increase up to the compliance current. The contact resistance (R) was calculated in this range using $R = \sum_{i=1}^{n} \frac{dV_i}{dI_i}/n$, were where n is the number of devices tested. We obtained $R = 11.8 \pm 3.9 \Omega$ and 12.0 ± 3.0 Ω for the PN and PIN devices, respectively. The contact resistance for devices with other lengths were similarly calculated. For the PIN devices, $R = 10.6 \pm 3.5$, 9.0 ± 3.3, and 8.7 ± 3.5 Ω for device lengths of 100, 200 and 500 µm, respectively; and for PN devices, the corresponding $R = 11.2 \pm 3.3$, 8.9 ± 2.3, and 9.7 ± 0.8 Ω.

C. RC-limited 3-dB bandwidth calculation

Here, we focus on 50-µm long devices. The RC-limited 3-dB OE bandwidth can be calculated using [5, 6]:

$$f_{RC} = \left[2\pi(R + 50\ \Omega)(C_j + C_p)\right]^{-1}, \quad \text{(S3)}$$

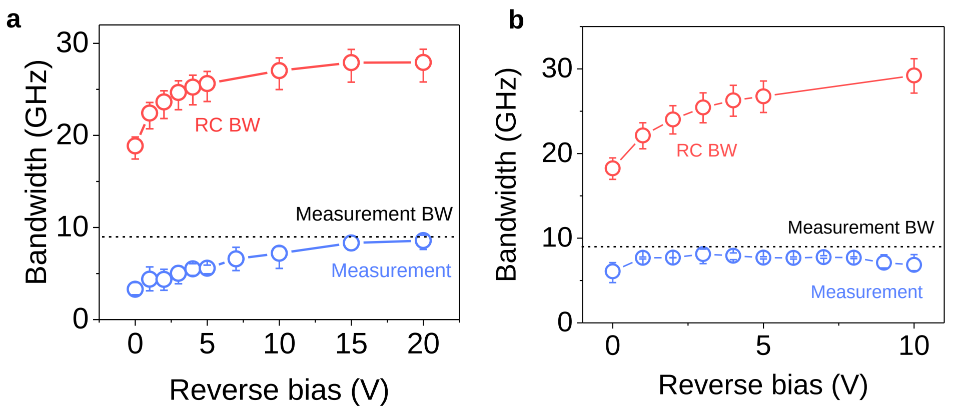

FIG. S16. Calculated RC-limited bandwidth compared with the measured 3-dB bandwidth for 50-µm long (a) PIN and (b) PN devices. Data were collected from 3 different chips on the wafer. The error bars show the maximum and minimum quantities measured for the 3 devices, and the data point shows the average.

where R, C_j, and C_p are the extracted values and 50 Ω is the load resistance from the measurement apparatus for impedance matching. Figure S16 shows the calculated f_{RC} as a function of reverse bias (red curves). For comparison, 3-dB bandwidths obtained from the fast Fourier transform of the impulse responses are included in the same plots (blue curves). The calculated RC-limited bandwidths are significantly higher than that from the impulse responses. In the measurements, the bandwidth saturated at ∼9 GHz, for both PIN and PN devices, which may be limited by the laser source (pulse FWHM ∼50 ps) as well as the oscilloscope bandwidths (∼13 GHz). Thus, for the PIN devices, at low V_r ($|V_r| < 10$ V), where the 3-dB bandwidth is < 5 GHz, the OE bandwidth is likely limited by the carrier transit time; while at high V_r ($|V_r| > 10$ V), the measurement instrumentation limits the OE bandwidth. For the PN devices, the 3-dB bandwidth is limited by the measurement instrumentations for $|V_r| > 1$ V. A more accurate measurement of the OE bandwidth would thus require a shorter input pulse width and a higher bandwidth oscilloscope.

S11. APD PARAMETERS FOR $l = 100$ μm

Figure S17 shows M, 3-dB OE bandwidth and GBP results for the 100-µm long devices. The method to extract the parameters is described in the Methods section of the main text.

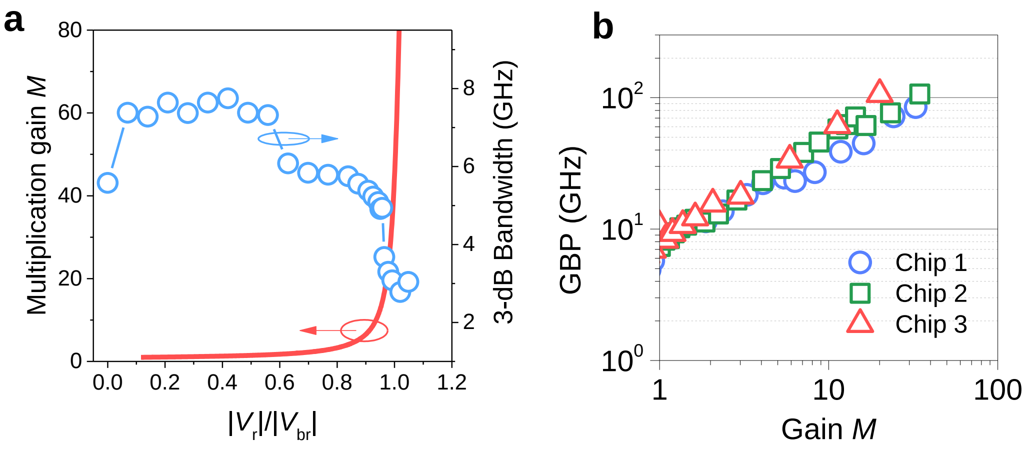

FIG. S17. (a) Avalanche multiplication gain (M) and 3-dB bandwidth for a PN device with $l = 100$ µm. (b) The corresponding GBP as a function of M for devices from 3 different chips on the wafer.

S12. V_{br} DETERMINATION

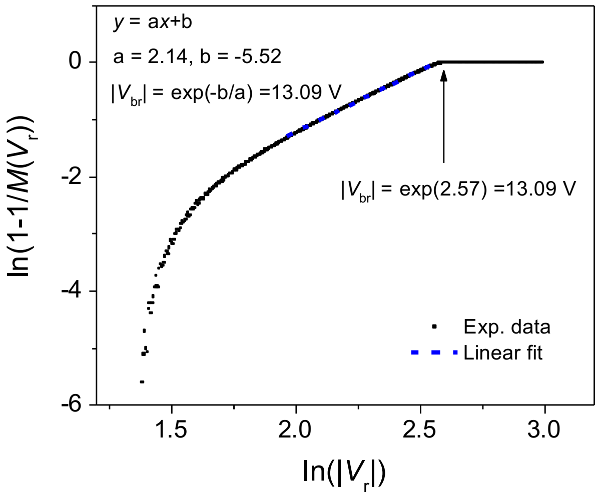

FIG. S18. V_{br} extraction via linear fitting and extrapolation.

Figure S18 shows an example plot of the fitting to determine V_{br} for a 50-µm long PN device, with a resultant $V_{br} = -13.1$ V. The result agrees well with the onset of the saturation of the curve (indicated by the arrow, $\ln(|V_r|) = 2.57$), beyond which the device clearly experienced an avalanche breakdown (i.e., $\ln\left(1 - \frac{1}{M(V_r)}\right) \sim 0$).

S13. COMPARISON WITH THE STATE OF THE ART

Table S2 compares this work with the state-of-the-art PDs integrated with SiN waveguides operating in the visible and near-infrared (NIR) range. A description of the comparison is in the main manuscript.

TABLE S2. Comparison of SiN waveguide-integrated visible and NIR (λ = 400-800 nm) PDs.

Device type	A (μm^2)	λ (nm)	I_{dark} (pA)	EQE (%)	BW (GHz)	M	GBP (GHz)	Ref.
SiN-on-Si mesa (PIN, PN)	24×50	400∼640	144±42@-5V* 266±65@-15V*	60 − 88 @-2V*	8.6±1.0 @-20V*	$46 \pm 14^{\dagger}$	$173 \pm 30^{\dagger}$	This work
End-fire SiN-on-SOI (PN)	$6^a \times 16$	685	<70@-2V	$\sim 40^a$	30	12.3	234±25	[7]
SOI-on-SiN (PIN)	11.6×200	775, 800	107@-3V	30	6	$\sim 10^a$	68	[8]
Al_2O_3-on-SOI (PIN)	N.A.×100	405	<1000	76	-	-	-	[9]
poly-Si (MSM)	1.14×10	654	200@-5V	67^a	-	-	-	[10]
α-Si (MSM)	30^a×50	660	25@4V, 50@8V	0.06^a	1×10^{-6}	-	-	[11]
$MoSe_2/WS_2$	$5^a \times 13$	780	50	158@-2V^a	0.02	-	-	[12]
AlGaAs/GaAs -on-Ta_2O_5	20×20 20×40	635	20@-2V	22.4	12.6	-	-	[13]

Legend: A: device active area (width×length); λ: operating wavelength range; I_{dark}: dark current; BW: 3-dB OE bandwidth at unity/low gain. *data from PIN devices; †data from PN devices; N.A.: not available; ^{a} The results were not explicitly reported but inferred from relevant data in literature.

[1] W. D. Sacher, X. Luo, Y. Yang, F.-D. Chen, T. Lordello, J. C. C. Mak, X. Liu, T. Hu, T. Xue, P. G.-Q. Lo, M. L. Roukes, and J. K. S. Poon, Visible-light silicon nitride waveguide devices and implantable neurophotonic probes on thinned 200 mm silicon wafers, Optics Express **27**, 37400 (2019).

[2] Y. Lin, J. C. C. Mak, H. Chen, X. Mu, A. Stalmashonak, Y. Jung, X. Luo, P. G.-Q. Lo, W. D. Sacher, and J. K. S. Poon, Low-loss broadband bi-layer edge couplers for visible light, Optics Express **29**, 34565 (2021).

[3] D. Ahn, L. C. Kimerling, and J. Michel, Evanescent coupling device design for waveguide-integrated group IV photodetectors, Journal of Lightwave Technology **28**, 3387 (2010).

[4] D. Ahn, C.-Y. Hong, L. C. Kimerling, and J. Michel, Coupling efficiency of monolithic, waveguide-integrated Si photodetectors, Applied Physics Letters **94**, 081108 (2009).

[5] J. Liu, J. Michel, W. Giziewicz, D. Pan, K. Wada, D. D. Cannon, S. Jongthammanurak, D. T. Danielson, L. C. Kimerling, J. Chen, F. Ö. Ilday, F. X. Kärtner, and J. Yasaitis, High-performance, tensile-strained Ge p-i-n photodetectors on a Si platform, Applied Physics Letters **87**, 103501 (2005).

[6] Y. Lin, K. H. Lee, S. Bao, X. Guo, H. Wang, J. Michel, and C. S. Tan, High-efficiency normal-incidence vertical p-i-n photodetectors on a germanium-on-insulator platform, Photonics Research **5**, 702 (2017).

[7] S. Yanikgonul, V. Leong, J. R. Ong, T. Hu, S. Y. Siew, C. E. Png, and L. Krivitsky, Integrated avalanche photodetectors for visible light, Nature Communications **12**, 1834 (2021).

[8] S. Cuyvers, A. Hermans, M. Kiewiet, J. Goyvaerts, G. Roelkens, K. V. Gasse, D. V. Thourhout, and B. Kuyken, Heterogeneous integration of Si photodiodes on silicon nitride for near-visible light detection, Optics Letters **47**, 937 (2022).

[9] R. Morgan, D. Kharas, J. Knecht, P. Juodawlkis, K. Cahoy, and C. Sorace-Agaskar, Waveguide-integrated blue light detector, in *2021 IEEE Photonics Conference (IPC)* (2021) pp. 1–2.

[10] G. Yuan, R. Pownall, P. Nikkel, C. Thangaraj, T. Chen, and K. Lear, Characterization of CMOS compatible waveguide-coupled leaky-mode photodetectors, IEEE Photonics Technology Letters **18**, 1657 (2006).

[11] C. De Vita, F. Toso, N. G. Pruiti, C. Klitis, G. Ferrari, M. Sorel, A. Melloni, and F. Morichetti, Amorphous-silicon visible-light detector integrated in silicon nitride waveguides, arXiv, 2202.04413 (2022).

[12] R. Gherabli, S. R. K. C. Indukuri, R. Zektzer, C. Frydendahl, and U. Levy, $MoSe_2/WS_2$ heterojunction photodiode integrated with a silicon nitride waveguide for visible light detection with high responsivity, arXiv, 2112.08920 (2021).

[13] M. Jafari, T. Fatema, D. R. Carlson, S. B. Papp, and A. Beling, Heterogeneous integration of AlGaAs/GaAs photodiodes on tantala waveguides for visible-light applications, in *Conference on Lasers and Electro-Optics* (2022) p. STu5G.5.
